# Racial/Ethnic Disparities in the Awareness, Treatment, and Control of Hypertension — United States, 2003–2010

**Published:** 2013-05-10

**Authors:** Amy L. Valderrama, Cathleen Gillespie, Carla Mercado

**Affiliations:** Div for Heart Disease and Stroke Prevention, National Center for Chronic Disease Prevention and Health Promotion; EIS Officer, CDC

Hypertension is a leading cause of cardiovascular disease and affects nearly one third of U.S. adults (1,2). Because the risk for cardiovascular disease mortality increases as blood pressure increases, clinical recommendations for persons with stage 2 hypertension (systolic blood pressure [SBP] ≥160 mmHg or diastolic blood pressure [DBP] ≥100 mmHg) include a more extensive treatment and follow-up regime than for those with stage 1 hypertension (SBP 140–159 mmHg or DBP 90–99 mmHg) (3). Although racial/ethnic disparities in the prevalence of hypertension have been well documented (4); ethnic disparities in the awareness, treatment, and control within blood pressure stages have not. To examine racial/ethnic disparities in awareness, treatment, and control of high blood pressure by hypertension stages, CDC analyzed data from the National Health and Nutrition Examination Survey (NHANES) for the period 2003–2010. This report describes the results of that analysis, which indicated that the proportion of Mexican-Americans and blacks with stage 1 and stage 2 hypertension was greater than for whites.* Among those with stage 1 hypertension, treatment with medication was significantly lower for Mexican-Americans compared with their non-Hispanic counterparts. Although treatment among persons with stage 2 hypertension did not differ by race/ethnicity, less than 60% of those with stage 2 hypertension were treated with medication. More efforts are needed to reduce barriers to accessing health care and low-cost medication, as well as increasing clinicians’ hypertension treatment knowledge and adherence to clinical guidelines.

NHANES is an ongoing, stratified, multistage probability sample of the noninstitutionalized U.S. civilian population.† Interviews and detailed physical examinations are performed. To obtain statistically stable estimates within racial/ethnic groups, CDC analyzed data from four 2-year cycles (2003–2010). Examination response rates ranged from 75% to 77% during this period, resulting in a total of 22,992 adult (aged ≥18 years) participants. The analysis excluded women who were pregnant (n = 732), participants without a blood pressure measurement (n = 1,339), other Hispanics and persons of other race or of multiple race (n = 2,693), and persons without hypertension (n = 14,313). Some participants were excluded based on more than one criterion, yielding a final study sample of 6,632 participants. Hypertension was defined as an average SBP ≥140 mmHg or DBP ≥90 mmHg, based on the average of up to three blood pressure measurements,§ or self-report of currently using blood pressure–lowering medication. Hypertension treatment was identified as the use of blood pressure–lowering medication and did not include lifestyle or dietary approaches. Hypertension stages were classified as stage 1 hypertension (SBP 140–159 mmHg or DBP 90–99 mmHg) and stage 2 hypertension (SBP ≥160 mmHg or DBP ≥100 mmHg) (3). Blood pressure control was defined as an SBP <140 mmHg and DBP <90 mmHg among those with hypertension. Hypertension awareness was determined based on whether a participant was ever told they had high blood pressure by a health-care provider. Health-care coverage was categorized into three groups: 1) Medicare, 2) private insurance, or 3) public insurance, which included Medicaid, a military health plan, or a state-sponsored plan.

All analyses were performed using statistical software to account for sampling weights and adjust variance estimates for the complex sampling design. A univariate chi-square test of independence was used to determine statistically significant (p<0.05) differences across racial/ethnic groups. Because multiple NHANES cycles were combined, trends over time could not be examined, and prevalence estimates could not be age adjusted. Population counts were estimated using the Current Population Surveys provided from NHANES by averaging the population during the period coinciding with the four NHANES cycles.¶

Among those with hypertension, the proportion of persons who were aged <65 years was greater for blacks (74.1%) and Mexican-Americans (71.9%) compared with whites (57.4%) (Table 1). Hypertension awareness, treatment, and control were lowest among Mexican-Americans (68.7%, 58.7%, and 35.5%, respectively) compared with whites (aware: 79.1%, treated: 71.2%, and controlled: 48.6%) and blacks (aware: 80.8%, treated: 71.9%, and controlled: 43.0%).

Among those with uncontrolled hypertension, awareness and treatment was greater for blacks (66.3% and 50.7%, respectively) compared with whites (aware: 59.4%, treated: 44.0%) and Mexican-Americans (aware: 51.4%, treated: 35.9%) (Table 2). Blacks with stage 1 hypertension had greater awareness (61.3%) and treatment (47.4%) compared with whites (awareness: 57.4%, treatment: 42.1%) and Mexican-Americans (awareness: 45.2%, treatment: 30.0%). Among those with stage 2 hypertension, blacks had greater awareness (77.6%) compared with whites (65.7%) and Mexican-Americans (66.0%); however, no difference was observed in hypertension treatment by race/ethnicity. Health-care coverage for those with uncontrolled hypertension was lowest for Mexican-Americans (59.3%) compared with blacks (77.7%) and whites (89.4%). However, among all persons with uncontrolled hypertension who were treated, the proportion who had health-care coverage was lower for Mexican-Americans (75.0%) compared with blacks (86.9%) and whites (94.4%). Awareness and treatment increased from stage 1 to stage 2 hypertension across all racial/ethnic groups.

## Editorial Note

The results presented in this report indicate that during 2003–2010, racial/ethnic disparities existed among U.S. adults with hypertension and within hypertension stages for age, awareness, treatment, and health-care coverage. Mexican-Americans and blacks with hypertension were significantly younger than whites. This might reflect earlier onset of hypertension among these racial/ethnic groups (5). Awareness and treatment was highest among blacks. This association is consistent with previous studies (6,7) and might be a result of efforts to reduce the persistent high prevalence of hypertension among blacks. Although no significant difference was observed in hypertension treatment by race/ethnicity among those with stage 2 hypertension, treatment was low overall (50%–58%) in this high-risk group, for whom clinical guidelines recommend a two-drug combination (3). Data on the number or type of medication used by participants, including two-drug combinations, were not examined in this report. A greater proportion of blood pressure control among those treated for hypertension has been observed among Mexican-Americans (74%) and whites (75%) compared with blacks (62%) (6). To improve treatment and achieve the *Healthy People 2020* goal of blood pressure control in 61.2% of persons with hypertension (8) across all race/ethnic groups, targeted implementation of demonstrated, evidence-based community and clinical strategies is necessary (1).

In this study, the proportion of persons with health-care coverage was lowest among Mexican-Americans. Lack of health-care coverage has been associated with lower rates of hypertension awareness, treatment, and control (9). This might partially explain the observed lower treatment and awareness of hypertension among Mexican-Americans in this report.

What is already known on this topic?

It has been previously reported that one in three U.S. adults had high blood pressure during 2009–2010, and approximately half (53.3%) had their condition under control. The prevalence of high blood pressure differs by race/ethnicity, with the condition being more common among blacks (40.4%) compared with whites (27.4%) and Mexican-Americans (26.1%).

What is added by this report?

Based on data from the National Health and Nutrition Examination Survey for the period 2003–2010, high blood pressure control differed for whites (48.6%), blacks (43.0%), and Mexican-Americans (35.5%). Among those with hypertension, the proportion with stage 2 hypertension was greater for Mexican-Americans (19.2%) and blacks (17.7%) compared with whites (12.3%).

What are the implications for public health practice?

To reduce the prevalence of uncontrolled high blood pressure and the associated racial/ethnic disparities, efforts are needed to increase hypertension awareness and hypertension treatment and adherence, especially in the Mexican-American population. The Million Hearts initiative focuses on addressing these issues by presenting a multifactorial approach focusing on reducing cardiovascular risk factors, such as high blood pressure, and tailoring this approach to effectively reach different racial/ethnic populations.

The findings in this report are subject to at least five limitations. First, although the focus of the study was to investigate racial/ethnic disparities within blood pressure stages, CDC did not consider other racial/ethnic groups or respondents who were multiracial because sample sizes were too small for meaningful analysis. Similarly, the study could not consider other Hispanic subpopulations or Hispanics as a whole because of differences in NHANES sample design between the 2003–2006 and 2007–2010 cycles. Second, hypertension awareness and treatment as well as other covariates were self-reported and subject to recall bias. Third, hypertension treatment was based only on medication use, not accounting for participants who were using lifestyle or dietary approaches to reduce blood pressure, which might have resulted in an underestimation of proportion of adults with hypertension who received “treatment.” Fourth, because of a limited number of participants with stage 2 hypertension within each cycle of NHANES, changes over time in the estimates were not evaluated. Finally, NHANES examination response rates ranged from 75% to 77%.

Racial/ethnic disparities exist in blood pressure, awareness, treatment, and control, with Mexican-Americans having a lower awareness and treatment of hypertension, as well as less health-care coverage, compared with blacks and whites. Multiple national efforts target improvements in high blood pressure prevention, treatment, and control (3). The Million Hearts initiative, co-led by CDC and the Centers for Medicare and Medicaid Services, is focusing efforts on preventing 1 million heart attacks and strokes by 2017, partially achieved by increasing blood pressure control for 10 million persons in the United States (10).** Million Hearts is working to reduce cardiovascular disease risk factors through parallel efforts aimed at clinical settings and communities with a focus on the “ABCS” (i.e., appropriate aspirin use for those at risk, blood pressure control, cholesterol management, and smoking cessation). The initiative aims to improve prescription and patient adherence to appropriate medications for the ABCS, promote a heart-healthy lifestyle, and refine access to effective care, while bringing clinicians’ attention to cardiovascular disease prevention, including appropriate drug regimens. Million Hearts also provides communities and clinical settings with resources and materials that are tailored for different racial/ethnic populations.

## Figures and Tables

**TABLE 1:** Prevalence of selected characteristics among adults aged ≥18 years with hypertension,* by race/ethnicity — National Health and Nutrition Examination Survey, United States, 2003–2010†

	Mexican-American				White, non-Hispanic				Black, non-Hispanic				
				
	N = 1,062				N = 3,766				N = 1,804				
				
Characteristic	Sample size	%	(95% CI)	No. in population (in millions)	Sample size	%	(95% CI)	No. in population (in millions)	Sample size	%	(95% CI)	No. in population (in millions)	p-value§
**Sex**													
Male	505	52.4	(49.4–55.4)	1.6	1,945	49.2	(47.6–50.7)	22.4	855	42.8	(40.4–45.2)	3.7	<0.001
Female	557	47.6	(44.6–50.6)	1.4	1,821	50.8	(49.3–52.4)	23.1	949	57.2	(54.8–59.6)	5.0	
**Age group (yrs)**													
18–44	121	25.0	(20.7–29.4)	0.8	370	13.4	(11.6–15.2)	6.1	284	21.4	(19.2–23.6)	1.9	<0.001
45–64	488	46.9	(43.3–50.6)	1.4	1,207	44.0	(42.1–46.0)	20.0	869	52.7	(50.4–55.0)	4.6	
≥65	453	28.0	(25.3–30.7)	0.8	2,189	42.6	(40.4–44.8)	19.4	651	25.9	(23.2–28.7)	2.2	
**Education (respondents aged ≥25 yrs)**													
Less than high school diploma	677	57.7	(52.7–62.8)	1.7	869	17.9	(15.2–20.6)	8.1	618	31.6	(28.5–34.7)	2.7	<0.001
High school diploma	170	19.3	(16.2–22.4)	0.6	1,116	29.9	(27.8–32.0)	13.5	431	24.8	(22.4–27.2)	2.1	
Some college	141	15.2	(11.4–19.0)	0.4	1,014	29.2	(27.2–31.2)	13.2	489	29.7	(27.5–31.9)	2.5	
College degree or higher	58	7.8	(5.3–10.3)	0.2	737	23.1	(20.4–25.7)	10.4	235	13.9	(12.0–15.9)	1.2	
**Poverty-to-income ratio** ¶													
<100%	298	27.0	(21.6–32.4)	0.8	403	7.2	(5.9– 8.6)	3.3	336	18.5	(16.1–20.8)	1.6	<0.001
100%–299%	474	43.1	(38.6–47.6)	1.3	1,642	36.8	(34.2–39.4)	16.8	795	43.7	(40.9–46.4)	3.8	
300%–499%	126	13.9	(10.6–17.2)	0.4	782	25.3	(23.0–27.6)	11.5	320	18.0	(15.7–20.4)	1.6	
≥500%	164	16.0	(11.9–20.1)	0.5	939	30.6	(27.6–33.6)	13.9	353	19.8	(17.6–22.0)	1.7	
**Hypertension awareness** **													
Aware	768	68.7	(64.9–72.4)	2.1	2,996	79.1	(77.3–80.9)	36.0	1,486	80.8	(78.2–83.4)	7.0	<0.001
Unaware	294	31.3	(27.6–35.1)	0.9	770	20.9	(19.1–22.7)	9.5	318	19.2	(16.6–21.8)	1.7	
**Hypertension treatment** ††													
Treated	674	58.7	(53.7–63.6)	1.8	2,725	71.2	(68.9–73.4)	32.4	1,335	71.9	(68.9–74.9)	6.2	<0.001
Untreated	386	41.3	(36.4–46.3)	1.2	1,035	28.8	(26.6–31.1)	13.1	469	28.1	(25.1–31.1)	2.4	
**Hypertension controlled** §§													
Yes	402	35.5	(32.7–38.3)	1.1	1,795	48.6	(46.3–50.8)	22.1	786	43.0	(40.3–45.7)	3.7	<0.001
No	660	64.5	(61.7–67.3)	1.9	1,971	51.4	(49.2–53.7)	23.4	1,018	57.0	(54.3–59.7)	4.9	
**Blood pressure stages** ¶¶													
Normal	127	12.0	(10.1–14.0)	0.4	660	17.8	(16.5–19.1)	8.1	286	16.5	(14.8–18.1)	1.4	<0.001
Pre-hypertension	275	23.5	(21.0–26.0)	0.7	1,135	30.8	(28.9–32.6)	14.0	500	26.5	(24.3–28.7)	2.3	
Stage 1 hypertension	435	45.3	(41.3–49.2)	1.4	1,429	39.2	(36.9–41.4)	17.8	699	39.3	(36.9–41.8)	3.4	
Stage 2 hypertension	225	19.2	(16.1–22.2)	0.6	542	12.3	(11.1–13.4)	5.6	319	17.7	(15.6–19.8)	1.5	
**Health-care coverage** ***													
No	302	35.0	(31.1–38.9)	1.1	289	8.1	(6.8– 9.3)	3.7	254	16.8	(14.5–19.0)	1.5	<0.001
Yes	760	65.0	(61.1–68.9)	2.0	3,477	91.9	(90.7–93.2)	41.8	1,550	83.2	(81.0–85.5)	7.2	
**Health-care coverage type** †††													
Medicare	204	19.6	(14.9–24.3)	0.4	645	13.0	(11.5–14.5)	5.4	280	14.3	(12.7–16.0)	1.0	<0.001
Private	344	53.3	(47.3–59.4)	1.0	2,215	72.1	(69.9–74.3)	30.2	874	59.3	(56.5–62.2)	4.3	
Public	212	27.0	(22.2–31.9)	0.5	617	14.9	(13.3–16.6)	6.2	396	26.4	(23.3–29.4)	1.9	
**Routine place for health care** §§§													
Yes	909	81.1	(78.1–84.0)	2.4	3,592	94.8	(93.9–95.7)	43.1	1,721	94.7	(93.4–95.9)	8.2	<0.001
No	153	18.9	(16.0–21.9)	0.6	174	5.2	(4.3– 6.1)	2.4	83	5.3	(4.1– 6.6)	0.5	
**No. of times received health care in past year** ¶¶¶													
0	151	18.0	(14.8–21.2)	0.5	190	5.5	(4.4– 6.7)	2.5	132	8.5	(7.1–10.0)	0.7	<0.001
1	139	14.8	(11.7–17.8)	0.4	387	12.3	(10.9–13.6)	5.6	181	10.5	(9.0–12.1)	0.9	
≥2	772	67.2	(62.8–71.6)	2.0	3,187	82.2	(80.6–83.8)	37.4	1,487	80.9	(79.2–82.7)	7.0	

**Abbreviation:** CI = confidence interval.

* Defined as systolic blood pressure (SBP) ≥140 mmHg or diastolic blood pressure (DBP) ≥90 mmHg or currently using blood pressure–lowering medication.

† Adult participants with no blood pressure measurement, self-reported race/ethnicity as “other/multiracial,” and pregnant women were excluded.

§ Pearson chi-squared statistic, corrected for survey design.

¶ Ratio of family income to poverty as defined by the U.S. Census Bureau. Information available at http://www.census.gov/hhes/www/poverty/methods/definitions.html#ratio of income to poverty.

** Based on responses to the following questions, “Have you ever been told by a doctor or other health-care professional that you had hypertension, also called high blood pressure?” and “Were you told on two or more different visits that you had hypertension or high blood pressure?”

†† Based on whether the participant answered “yes” to both of the following questions: “Because of your high blood pressure, have you ever been told to take prescribed medicine?” and “Are you now taking prescribed medicine for high blood pressure?”

§§ Based on blood pressure measurements for those with hypertension: controlled (SBP <140 and DBP <90) and uncontrolled (SBP ≥140 or DBP ≥90).

¶¶ Classified as normal (SBP <120 and DBP <80), pre-hypertension (SBP 120–139 or DBP 80–89), stage 1 hypertension (SBP 140–159 or DBP 90–99), and stage 2 hypertension (SBP ≥160 or DBP ≥100).

*** Participants were asked, “Are you covered by health insurance or some other health-care plan?”

††† Health-care coverage types reported were Medicare, private insurance, and/or public health insurance (Medicaid, Children’s Health Insurance Program [CHIP], state or other government sponsored health plan, or military health plan).

§§§ Based on response to the question, “Is there a place that you usually go when sick or need advice about health?”

¶¶¶ Based on response to the question, “During the past 12 months, how many times have you seen a doctor or other health-care professional about your health, not including being hospitalized overnight?”

**TABLE 2:** Prevalence of selected characteristics among adults aged ≥18 years with uncontrolled hypertension,* by stage of hypertension† — National Health and Nutrition Examination Survey, United States, 2003–2010

	All uncontrolled hypertension												
		
	Mexican-American (n = 660)				White, non-Hispanic (n = 1,971)				Black, non-Hispanic (n = 1,018)				
				
Characteristic	Sample size	%	(95% CI)	No. in population (in millions)	Sample size	%	(95% CI)	No. in population (in millions)	Sample size	%	(95% CI)	No. in population (in millions)	p-value§
**Sex**													
Male	321	53.9	(49.7–58.2)	1.0	1,009	49.9	(47.9–51.8)	11.7	523	48.0	(44.9–51.2)	2.4	0.139
Female	339	46.1	(41.8–50.3)	0.9	962	50.1	(48.2–52.1)	11.7	495	52.0	(48.8–55.1)	2.6	
**Age group (yrs)**													
18–44	97	31.8	(27.1–36.5)	0.6	218	14.9	(12.5–17.3)	3.5	197	25.4	(22.3–28.4)	1.3	<0.001
45–64	271	39.6	(34.8–44.5)	0.8	587	42.0	(39.4–44.7)	9.8	477	50.4	(47.6–53.1)	2.5	
≥65	292	28.6	(24.8–32.3)	0.6	1,166	43.0	(40.3–45.8)	10.1	344	24.3	(21.0–27.5)	1.2	
**Hypertension awareness** ¶													
Aware	366	51.4	(46.8–56.0)	1.0	1,201	59.4	(56.7–62.0)	13.9	700	66.3	(62.6–70.1)	3.3	<0.001
Unaware	294	48.6	(44.0–53.2)	0.9	770	40.6	(38.0–43.3)	9.5	318	33.7	(29.9–37.4)	1.7	
**Hypertension treatment** **													
Treated	274	35.9	(30.1–41.7)	0.7	936	44.0	(41.3–46.7)	10.3	549	50.7	(46.6–54.8)	2.5	0.001
Untreated	386	64.1	(58.3–69.9)	1.2	1,035	56.0	(53.3–58.7)	13.1	469	49.3	(45.2–53.4)	2.4	
**Health-care coverage** ††													
Yes	441	59.3	(55.1–63.5)	1.2	1,783	89.4	(87.6–91.1)	20.9	825	77.7	(74.7–80.7)	3.8	<0.001
No	219	40.7	(36.5–44.9)	0.8	188	10.6	(8.9–12.4)	2.5	193	22.3	(19.3–25.3)	1.1	
**Routine place for health care** §§													
Yes	524	73.5	(69.1–77.9)	1.4	1,824	91.4	(89.7–93.2)	21.4	946	91.9	(89.7–94.1)	4.5	<0.001
No	136	26.5	(22.1–30.9)	0.5	147	8.6	(6.8–10.3)	2.0	72	8.1	(5.9–10.3)	0.4	
**No. of times received health care in past year** ¶¶													
0	136	25.5	(21.6–29.4)	0.5	175	10.0	(7.9–12.0)	2.3	125	14.2	(11.7–16.7)	0.7	<0.001
1	109	18.6	(14.5–22.7)	0.4	253	15.8	(14.1–17.6)	3.7	135	13.7	(11.1–16.3)	0.7	
≥2	415	55.9	(51.0–60.8)	1.1	1,542	74.2	(71.9–76.5)	17.4	757	72.1	(69.0–75.2)	3.6	

	Stage 1 hypertension												
		
	Mexican-American (n = 435)				White, non-Hispanic (n = 1,429)				Black, non-Hispanic (n = 699)				
				
Characteristic	Sample size	%	(95% CI)	No. in population (in millions)	Sample size	%	(95% CI)	No. in population (in millions)	Sample size	%	(95% CI)	No. in population (in millions)	p-value§
**Sex**													
Male	227	57.1	(50.7–63.4)	0.8	779	52.9	(50.5–55.3)	9.4	381	50.4	(46.5–54.2)	1.7	0.212
Female	208	42.9	(36.6–49.3)	0.6	650	47.1	(44.7–49.5)	8.4	318	49.6	(45.8–53.5)	1.7	
**Age group (yrs)**													
18–44	81	37.4	(31.3–43.5)	0.5	190	17.3	(14.5–20.2)	3.1	157	28.3	(24.5–32.2)	1.0	<0.001
45–64	184	38.7	(32.8–44.6)	0.5	478	45.0	(41.9–48.0)	8.0	333	50.8	(47.0–54.5)	1.7	
≥65	170	23.9	(19.7–28.1)	0.3	761	37.7	(35.0–40.4)	6.7	209	20.9	(16.8–25.0)	0.7	
**Hypertension awareness** ¶													
Aware	212	45.2	(40.5–50.0)	0.6	839	57.4	(54.3–60.5)	10.2	450	61.3	(57.4–65.2)	2.1	<0.001
Unaware	223	54.8	(50.0–59.5)	0.7	590	42.6	(39.5–45.7)	7.6	249	38.7	(34.8–42.6)	1.3	
**Hypertension treatment** **													
Treated	153	30.0	(24.6–35.4)	0.4	644	42.1	(39.1–45.2)	7.5	358	47.4	(43.5–51.4)	1.6	<0.001
Untreated	282	70.0	(64.6–75.4)	1.0	785	57.9	(54.8–60.9)	10.3	341	52.6	(48.6–56.5)	1.8	
**Health-care coverage** ††													
Yes	287	58.4	(53.5–63.4)	0.8	1,279	88.9	(86.9–90.8)	15.8	576	79.4	(76.1–82.6)	2.7	<0.001
No	148	41.6	(36.6–46.5)	0.6	150	11.1	(9.2–13.1)	2.0	123	20.6	(17.4–23.9)	0.7	
**Routine place for health care** §§													
Yes	342	72.7	(66.9–78.5)	1.0	1,314	91.0	(88.9–93.0)	16.2	650	92.1	(89.7–94.4)	3.1	<0.001
No	93	27.3	(21.5–33.1)	0.4	115	9.0	(7.0–11.1)	1.6	49	7.9	(5.6–10.3)	0.3	
**No. of times received health care in past year** ¶¶													
0	95	26.9	(22.3–31.4)	0.4	128	10.0	(7.8–12.1)	1.8	80	13.7	(11.0–16.4)	0.5	<0.001
1	75	18.9	(14.2–23.6)	0.3	194	16.4	(14.3–18.4)	2.9	96	14.3	(11.3–17.3)	0.5	
≥2	265	54.2	(48.2–60.2)	0.7	1,106	73.6	(71.1–76.2)	13.1	522	72.0	(68.7–75.3)	2.5	

	Stage 2 hypertension												
		
	Mexican-American (n = 225)				White, non-Hispanic (n = 542)				Black, non-Hispanic (n = 319)				
				
Characteristic	Sample size	%	(95% CI)	No. in population (in millions)	Sample size	%	(95% CI)	No. in population (in millions)	Sample size	%	(95% CI)	No. in population (in millions)	p-value§
**Sex**													
Male	94	46.6	(39.9–53.2)	0.3	230	40.1	(36.3–44.0)	2.2	142	42.8	(37.0–48.5)	0.7	0.310
Female	131	53.4	(46.8–60.1)	0.3	312	59.9	(56.0–63.7)	3.3	177	57.2	(51.5–63.0)	0.9	
**Age group (yrs)**													
18–44	16	18.7	(11.4–26.1)	0.1	28	7.1	(4.0–10.3)	0.4	40	18.7	(13.4–24.1)	0.3	<0.001
45–64	87	41.8	(34.6–49.0)	0.2	109	32.7	(28.4–37.0)	1.8	144	49.5	(44.1–54.9)	0.8	
≥65	122	39.5	(32.2–46.8)	0.2	405	60.2	(55.5–64.8)	3.4	135	31.8	(26.9–36.6)	0.5	
**Hypertension awareness** ¶													
Aware	154	66.0	(55.7–76.2)	0.4	362	65.7	(61.6–69.7)	3.7	250	77.6	(71.8–83.4)	1.2	0.010
Unaware	71	34.0	(23.8–44.3)	0.2	180	34.3	(30.3–38.4)	1.9	69	22.4	(16.6–28.2)	0.3	
**Hypertension treatment** **													
Treated	121	49.9	(39.6–60.3)	0.3	292	49.9	(44.9–54.9)	2.8	191	58.0	(51.0–65.0)	0.9	0.163
Untreated	104	50.1	(39.7–60.4)	0.3	250	50.1	(45.1–55.1)	2.8	128	42.0	(35.0–49.0)	0.6	
**Health-care coverage** ††													
Yes	154	61.3	(54.2–68.4)	0.4	504	90.9	(87.5–94.3)	5.1	249	74.1	(68.5–79.7)	1.1	<0.001
No	71	38.7	(31.6–45.8)	0.2	38	9.1	(5.7–12.5)	0.5	70	25.9	(20.3–31.5)	0.4	
**Routine place for health care** §§													
Yes	182	75.4	(68.3–82.5)	0.4	510	92.8	(90.3–95.2)	5.2	296	91.6	(87.2–96.0)	1.4	<0.001
No	43	24.6	(17.5–31.7)	0.1	32	7.2	(4.8–9.7)	0.4	23	8.4	(4.0–12.8)	0.1	
**No. of times received health care in past year** ¶¶													
0	41	22.3	(16.7–27.9)	0.1	47	10.0	(7.0–13.0)	0.6	45	15.3	(11.2–19.4)	0.2	0.005
1	34	17.9	(10.8–24.9)	0.1	59	14.0	(9.6–18.3)	0.8	39	12.5	(8.8–16.1)	0.2	
≥2	150	59.8	(53.9–65.8)	0.3	436	76.0	(71.1–80.9)	4.2	235	72.3	(66.7–77.9)	1.1	

**Abbreviation:** CI = confidence interval.

* Defined as an average systolic blood pressure (SBP) ≥140 mmHg or diastolic blood pressure (DBP) ≥90 mmHg.

† Stages of hypertension were stage 1 hypertension (SBP 140-159 or DBP 90-99) and stage 2 hypertension (SBP ≥160 or DBP ≥100).

§ Pearson chi-squared statistic, corrected for survey design.

¶ Based on responses to the following questions, “Have you ever been told by a doctor or other health-care professional that you had hypertension, also called high blood pressure?” and “Were you told on two or more different visits that you had hypertension or high blood pressure?”

** Based on whether the participant answered “yes” to both of the following questions: “Because of your high blood pressure, have you ever been told to take prescribed medicine?” and “Are you now taking prescribed medicine for high blood pressure?”

†† Participants were asked, “Are you covered by health insurance or some other health-care plan?”

§§ Based on response to the question, “Is there a place that you usually go when sick or need advice about health?”

¶¶ Based on response to the question, “During the past 12 months, how many times have you seen a doctor or other health-care professional about your health, not including being hospitalized overnight?”

## Footnotes

* For this report, all persons of black or white race are non-Hispanic. Mexican-Americans might be of any race.

† Additional information available at http://www.cdc.gov/nchs/nhanes.htm.

§ Additional information available at http://www.cdc.gov/nchs/nhanes.htm.

¶ Additional information available at http://www.cdc.gov/nchs/nhanes/response_rates_cps.htm.

** Additional information available at http://millionhearts.hhs.gov/index.html.
